# Case Report: Lacosamide unmasking *SCN5A*-associated Brugada syndrome in a young female with epilepsy

**DOI:** 10.3389/fcvm.2024.1406614

**Published:** 2024-05-31

**Authors:** Ying-Chi Shen, Jen-Chueh Wu, Ting-Tse Lin, Kai-Chung Chang, Jen-Jen Su, Jyh-Ming Jimmy Juang

**Affiliations:** ^1^ Department of Neurology, National Taiwan University Hospital, Taipei, Taiwan; ^2^ Department of Internal Medicine, National Taiwan University Hospital, Taipei, Taiwan; ^3^ Cardiovascular Center and Division of Cardiology, Department of Internal Medicine, National Taiwan University Hospital and National Taiwan University College of Medicine, Taipei, Taiwan; ^4^ Cardiovascular Center, Center of Heart Failure and Center of Genetic Heart Diseases, Division of Cardiology, Department of Internal Medicine, National Taiwan University Hospital and National Taiwan University College of Medicine, Taipei, Taiwan

**Keywords:** arrhythmia, Brugada syndrome, epilepsy, lacosamide, *SCN5A*, seizure

## Abstract

**Background:**

Lacosamide is frequently used as a mono- or adjunctive therapy for the treatment of adults with epilepsy. Although lacosamide is known to act on both neuronal and cardiac sodium channels, potentially leading to cardiac arrhythmias, including Brugada syndrome (BrS), its adverse effects in individuals with genetic susceptibility are less understood.

**Case:**

We report a 33-year-old female with underlying epilepsy who presented to the emergency department with a four-day history of seizure clusters, and was initially treated with lacosamide therapy. During the intravenous lacosamide infusion, the patient developed sudden cardiac arrest caused by ventricular arrhythmias necessitating resuscitation. Of note, the patient had a family history of sudden cardiac death. Workup including routine laboratory results, 12-lead electrocardiogram (ECG), echocardiogram, and coronary angiogram was non-specific. However, a characteristic type 1 Brugada ECG pattern was identified by ajmaline provocation testing; thus, confirming the diagnosis of BrS. Subsequently, the genotypic diagnosis was confirmed by Sanger sequencing, which revealed a heterozygous mutation (c.2893C>T, p.Arg965Cys) in the *SCN5A* gene. Eventually, the patient underwent implantable cardioverter-defibrillator implantation and was discharged with full neurological recovery.

**Conclusion:**

This case highlights a rare but lethal adverse event associated with lacosamide treatment in patients with genetic susceptibility. Further research is warranted to investigate the interactions between lacosamide and *SCN5A* variants.

## 1 Introduction

Lacosamide is a third-generation anti-seizure medication (ASM) characterized by enhancing slow-inactivated state of neuronal voltage-gated sodium channels (1). It is commonly used in adult patients for the treatment of seizure emergencies, such as status epilepticus or seizure clusters, because of its favorable safety profile, tolerability, and feasibility with intravenous (IV) administration (2, 3). Lacosamide also acts on cardiac sodium channels, which can potentially trigger cardiac arrhythmias, especially in patients who already have a predisposition, whether it be genetic, such as patients with Brugada syndrome (BrS), or of another type (4).

BrS is an inherited cardiac channelopathy associated with malignant arrhythmias and sudden cardiac death in young adults with structurally normal hearts, and is particularly prevalent in Asian populations (5). Notably, BrS is more commonly diagnosed in middle-aged males, typically at around 40 years of age. The incidence of BrS varies among different populations, showing marked genetic heterogeneity and geographical differences (6–8). Prior studies have estimated the prevalence of BrS at 12/10,000 in Southeast Asia, with a much lower rate of ∼5/10,000 in Western countries (9–10). The diagnostic hallmark of BrS is the presence of a type 1 Brugada electrocardiogram (ECG) pattern, which exhibits coved ST-segment elevation, followed by a negative T wave in the right precordial leads V1–V3. This specific ECG finding can occur spontaneously or be provoked by a drug provocation test using sodium channel blockers, such as ajmaline (5). Although BrS can be inherited in an autosomal dominant pattern, patients may present with diverse phenotypes owing to incomplete penetrance and variable expression, leading to diagnostic challenges (5).

Here, we report a unique case of a young lady with epilepsy who was resuscitated from sudden cardiac arrest during lacosamide therapy due to refractory ventricular arrhythmias, and was subsequently diagnosed as *SCN5A*-associated BrS.

## 2 Case presentation

A 33-year-old Indonesian woman, who worked as a live-in caretaker in Taiwan, initially presented to the neurological outpatient department with recurrent episodes of syncope with an upward eye deviation, which had been noticed over a period of two years. The routine awake electroencephalogram (EEG) revealed focal epileptiform discharges over the left temporal region (Supplementary Figure S1), suggesting epileptic seizure. No intracranial abnormalities were detected on the brain magnetic resonance imaging, and there were no abnormal laboratory findings. Initially, she was prescribed levetiracetam at a dose of 500 mg twice daily, but later switched to oxcarbazepine 300 mg twice daily due to intolerable dizziness. After ASM adjustment, she remained seizure-free for the following one year. However, she reported poor adherence to oxcarbazepine over the past one month due to financial issues. Three days before admission, she presented to another hospital with a head injury attributed to an unwitnessed fall at home. She was initially found unconscious on the ground by her employer, but regained consciousness upon arrival at the hospital. A head computed tomography scan revealed no abnormalities, and she was discharged. However, in the following hours, she experienced frequent episodes of sudden loss of consciousness, followed by bilateral hand twitching lasting for a few minutes, occasionally witnessed by her employer. Although she regained consciousness within five minutes, she had difficulty recalling the details of these events. Due to an increase in the frequency of episodic unconsciousness to six times a day, she was brought to our emergency department (ED) for medical attention.

**Figure 1:**
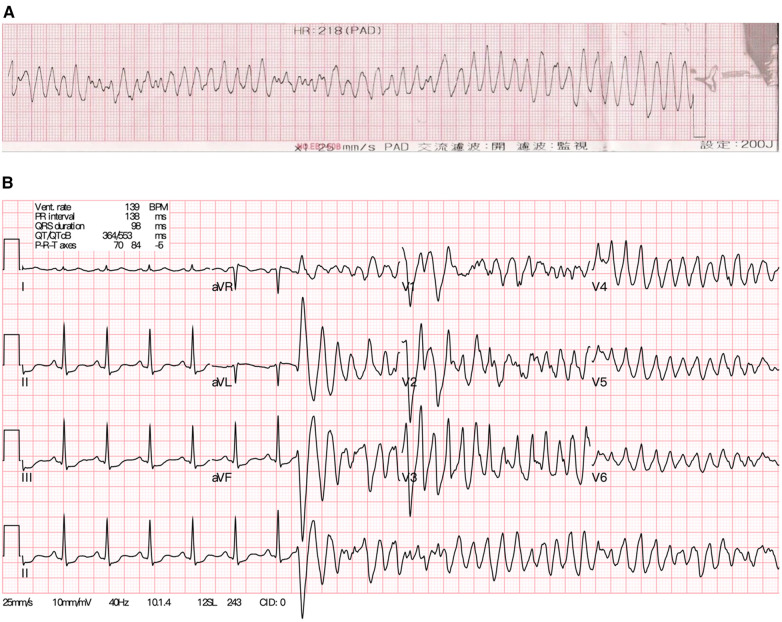
(**A**) The electrocardiogram of the young female patient showing ventricular fibrillation during resuscitation initially. (**B**) After achieving recovery of spontaneous circulation, the sinus rhythm soon degenerated into polymorphic ventricular tachycardia again.

Upon arrival at the ED, the patient was observed to have clonic movements in both hands, accompanied by upward gazing and impaired consciousness, lasting approximately two minutes. However, before receiving an IV push of lorazepam (2 mg) as prescribed by the ED physician, she gradually regained consciousness, albeit experiencing mild dizziness. In response to the suspicion of convulsive status epilepticus, IV treatment of ASM was initiated. A single IV loading dose of lacosamide (200 mg) was administered in 100 ml of normal saline, infused at a rate of 200 ml/h over 30 min. The ECG recorded on ED admission before lacosamide treatment was unremarkable. However, during the IV infusion of half the loading dose of lacosamide, the patient was found to be cyanotic and pulseless. As such, lacosamide was immediately discontinued due to concerns about its adverse cardiovascular effects. Cardiopulmonary resuscitation was initiated, with initial rhythm exhibiting ventricular fibrillation (VF) (Figure 1A). After two rounds of cardiac defibrillations, a transient return of spontaneous circulation was achieved. However, the cardiac rhythm of the patient degenerated into polymorphic ventricular tachycardia (VT) (Figure 1B), requiring further cardiac defibrillations. Venoarterial extracorporeal membrane oxygenation (V-A ECMO) was established as hemodynamic support. Concurrently, myoclonic jerks involving the left limbs with the head turning to the right were noted. Therefore, IV levetiracetam was administered for suspected focal motor seizures. Anti-arrhythmic drugs such as amiodarone, lidocaine and esmolol, in addition to deep sedation and magnesium sulfate were subsequently administered. Emergent coronary angiography revealed patent coronary arteries. Nevertheless, during the procedure, VT/VF persisted, despite repeated defibrillations. Therefore, a temporary transvenous pacemaker was placed for overdrive pacing.

**Figure 2:**
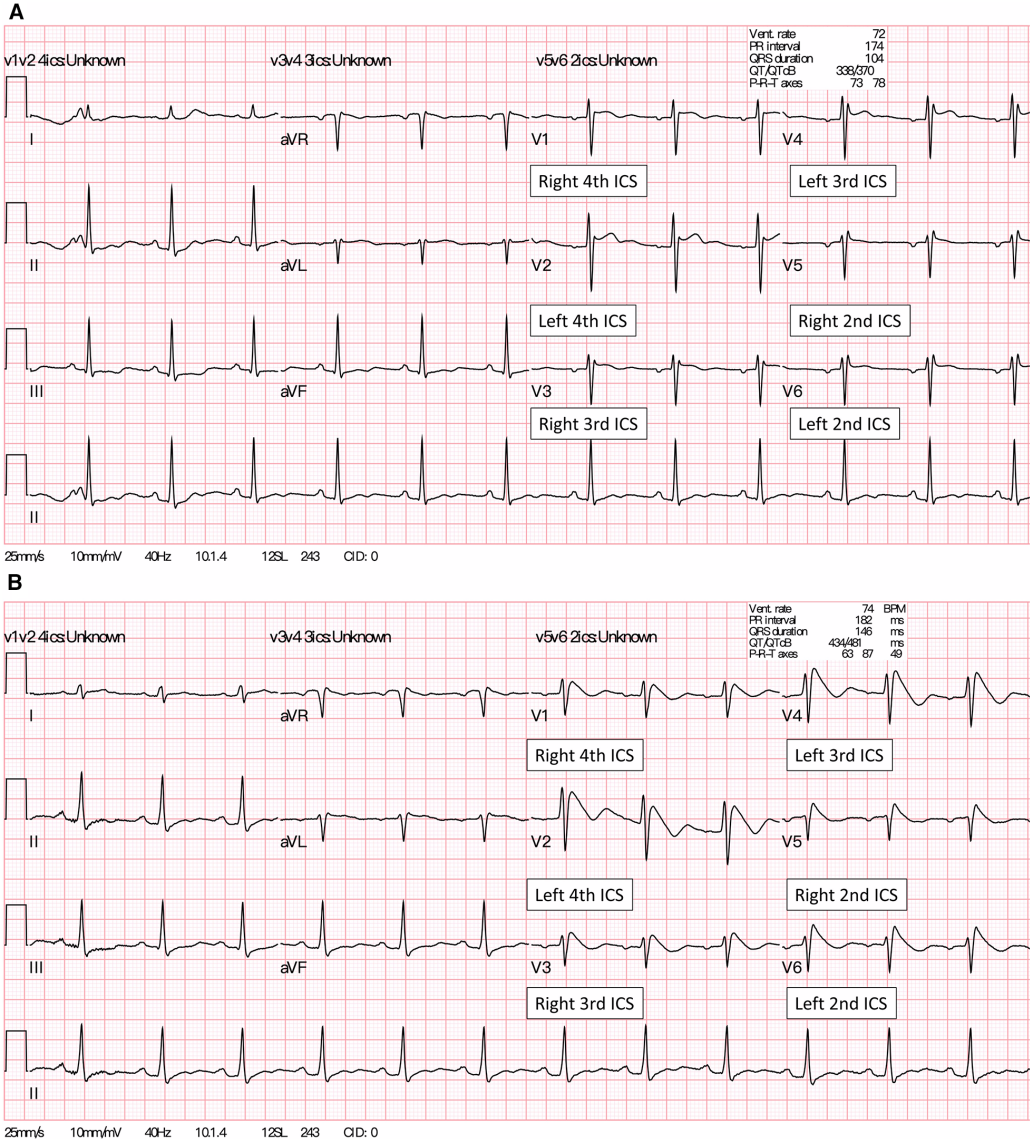
(**A**) A baseline 12-lead ECG of the patient showed a Brugada type 2 pattern (saddleback ST-segment elevation) in the precordial leads V1 placed at 4th ICS and lead V2 placed at 4th ICS. (**B**) About four minutes after the IV ajmaline injection, a Brugada type 1 pattern (coved-type ST-segment elevation) appeared in all precordial and high intercostal leads (V3-V6). Of note, lead V3 at the right 3rd ICS and lead V4 were placed at the left 3rd ICS, whereas lead V5 at right 2nd ICS and lead V6 were placed at the left 2nd ICS, respectively. ICS, intercostal space.

After transfer to the intensive care unit (ICU), the ventricular arrhythmias disappeared, and her hemodynamic parameters gradually stabilized. A comprehensive workup, including electrolytes, thyroid function, drug screening of blood and urine, as well as transthoracic echocardiography, was unremarkable. The testing of lacosamide plasma concentrations was not available at our hospital. Continuous EEG monitoring in the ICU after discontinuing sedative agents revealed focal interictal epileptiform discharges in the left frontal region, leading to the diagnosis of focal impaired awareness motor seizure. Afterwards, the patient regained consciousness at baseline level, and was weaned off the V-A ECMO on the fourth day of admission. Notably, although there was no positive family history of epilepsy, the patient reported that three relatives had experienced sudden cardiac death before the age of 40 years; hence, BrS was suspected. After tapering the overdrive pacing, repeat ECGs showed saddleback ST segment elevation in precordial leads V1–V3, suggestive a Brugada type 2 pattern (Figure 2A). Following this finding, an ajmaline drug provocation test was performed in the ICU. Serial ECGs showed a typical Brugada type 1 ECG pattern in the pericardial standard and high intercostal leads (Figure 2B), thus confirming the diagnosis of BrS in this young female patient. Subsequently, the patient underwent implantable cardioverter-defibrillator implantation and was discharged with full neurological recovery. Since the patient was a migrant worker, she was referred back to Indonesia for further medical care.

## 3 Analysis of genetic variants

Because of the significant family history of sudden death in this young adult and refractory ventricular arrhythmias following the use of sodium blocker, genetic variants in *SCN5A*, the most common gene associated with BrS, were screened by Sanger sequencing. Eventually, a heterozygous missense variant NM_198056.3: c.2893C>T (p. R965C) in exon 17 of *SCN5A* gene was identified (Supplementary Figure S2). The detected variant was previously described in ClinVar (variation ID: 67763). While *SCN5A* p.R965C variant is rare (minor allele frequency = 0.00001) in the general population database (gnomAD-ALL), its frequency is relatively higher in Asian populations, particularly enriched in the Thai population (0.00188), possibly as a result of the funder effect (11, 12). Its deleterious effect as predicted by silico predictions, conservation analysis and published functional studies (13). This variant was thus classified as likely pathogenic, according to the following American College of Medical Genetics and Genomics (ACMG) criteria (14): PS3, PP2, PP3, and BS1. Consequently, the patient was diagnosed with *SCN5A* mutation associated with BrS and epilepsy.

## 4 Discussion

We report a unique case of lacosamide unmasking *SCN5A*-associated BrS in a young Indonesian female with epilepsy. Mutations in *SCN5A*, which encodes for the alpha subunit of the cardiac voltage-gated sodium channel (Nav1.5), are the predominant genotype in BrS, accounting for 80% of all genotype-positive patients (15). In addition to the cardiac sodium channel, *SCN5A* is expressed on neuronal sodium channels in mouse and human brains (16). The dysfunction of *SCN5A* has been associated with epileptic seizure and increased risk of sudden unexpected death in epilepsy (17). Although several studies have identified the *SCN5A* mutation in patients with epilepsy co-existing with cardiac arrhythmias (18, 19), the c.2893C>T (p. R965C) mutation in *SCN5A* has not been reported in patients with seizure disorders.

In our case, it can be challenging to differentiate between seizure-like syncope and convulsive syncope secondary to inherited cardiac arrhythmias at the beginning of diagnosis, particularly if in the absence of EEG recording. Nevertheless, the diagnosis of focal impaired awareness seizure before the occurrence of lacosamide-related arrhythmia was confirmed in our patient based on the focal interictal epileptiform discharges recorded on the initial EEG, as well as a good response to ASM. Furthermore, the presence of focal epileptogenicity was also noticed on the continuous EEG monitoring after the patient experienced refractory ventricular tachyarrhythmias triggered by lacosamide. Therefore, the diagnosis of epileptic seizure in our patient could be ascertained. However, we did not perform continuous EEG monitoring concurrently with the ajmaline drug provocation test that triggered the Brugada type 1 pattern, so the direct impact of this variant on epilepsy phenotype was uncertain. More studies are required to analyze the genotype and phenotype of epilepsy caused by R965C mutation in *SCN5A*.

Hsueh et al. conducted an electrophysiological study of *SCN5A* p.R965C in a Taiwanese patient with BrS, demonstrating that this variant caused a loss of function in the cardiac sodium channel. This loss of function was attributed to a left shift of steady-state inactivation curve and slower recovery from inactivation of the sodium channel, resulting in a decrease in sodium current (13). When taking this study into account, *SCN5A* p.R965C variant would have been considered a deleterious mutation associated with BrS due to its alterations to channel functions. Prior studies have revealed ancestral differences in *SCN5A* p. R965C variants (11, 20). Compared to non-Asian ancestries, the allele frequency of the *SCN5A* p. R965C variant is higher in East Asian (0.00038, gnomAD) and South Asian (0.00006, gnomAD) (12, 20). Walsh et al. reported this variant as a rare non-coding enhancer variant in *SCN5A* in Thai patients with BrS (12). Although data for Indonesia populations remains uncertain, it is noteworthy that the highest frequency of this variant is observed in individuals of Thai and Malay ancestry, suggesting a significant enrichment in Southeast Asian populations (12). Taken together, our case supported the previous report and implied that the *SCN5A* p. R965C variant is not just a likely pathogenic variant but also a risk factor specifically for Asian patients with BrS (11, 12).

While the arrhythmogenic potential of lacosamide has previously been published (21), Goodnough et al. reported one case of lacosamide-induced Brugada ECG pattern. The patient was an 83-year-old man with septicemia who had previously received a six-month lacosamide treatment for a seizure disorder (4). In contrast, our young female patient presented with refractory VT/VF triggered by a single lacosamide infusion, without other concurrent triggering factors such as fever or infection. This difference may imply varying etiologies of lacosamide-induced BrS. The previous case may be attributed to acquired causes, while our patient's condition was related to inherited channelopathy. Furthermore, we had performed target genetic testing using Sanger sequencing, which increased diagnostic accuracy of inherited BrS.

Lacosamide is characterized by its stronger and faster binding to the slow-inactivated state compared to the fast-inactivated state, which may contribute to infrahisian conduction delay and QRS prolongation (1). In patients with *SCN5A* mutations, the use of lacosamide may cause the augmentation of slow inactivation of cardiac sodium channels, potentially leading to re-entrant arrhythmias and early afterdepolarizations in ventricular cardiac tissue (1, 21), which greatly increases the risk of refractory ventricular tachyarrhythmias and sudden cardiac arrest. In vitro, lacosamide has been shown to exert a concentration-dependent inhibitory effect on the peak amplitude of sodium currents in human embryonic kidney (HEK293T) cells expressing *SCN5A* (22). Nevertheless, further functional studies are warranted to explore the impact of lacosamide on *SCN5A* p.R965C variants. Interestingly, our patient receiving long-term treatment with oxcarbazepine, another sodium channel blocker, without triggering symptomatic cardiac conduction disorders, probably due to the lower serum plasma drug levels achieved by parenteral administration. However, considering the concentration-dependent inhibitory effect of lacosamide on sodium currents, a single IV loading of lacosamide may rapidly increase he serum concentration, potentially increasing the risk of VT/VF.

In conclusion, this case highlights that lacosamide can cause fatal ventricular tachyarrhythmias in patients with *SCN5A* mutation. Clinicians should be aware of these rare adverse effects when treating epileptic patients with lacosamide, particularly in Asia populations. Further research is warranted to investigate the interaction between lacosamide and *SCN5A* variants.

## Data Availability

The original contributions presented in the study are included in the article/Supplementary Material, further inquiries can be directed to the corresponding author.
