# Supplementary material for: Case Report: Lacosamide unmasking SCN5A-associated Brugada syndrome in a young female with epilepsy
Source: Front Cardiovasc Med. 2024 May 31;11:1406614. doi: 10.3389/fcvm.2024.1406614 (PMC11176425; doi:10.3389/fcvm.2024.1406614)
Supplement: Supplementary file 1 [file Image1.pdf]

Supplementary Material

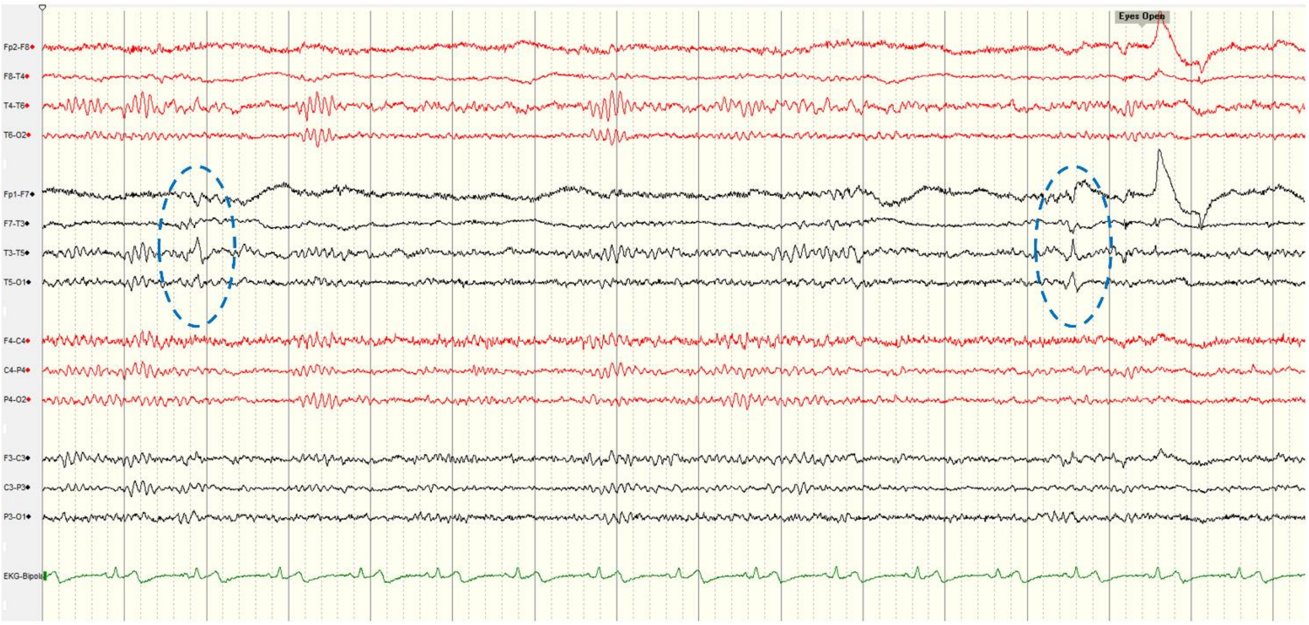

Supplementary Figure 1. EEG with longitudinal bipolar montage revealing interictal epileptiform discharges with focal sharp waves and phase reversals at T3 (the blue dashed circles). EEG, electroencephalogram.
