# Supplementary material for: Case Report: Lacosamide unmasking SCN5A-associated Brugada syndrome in a young female with epilepsy
Source: Front Cardiovasc Med. 2024 May 31;11:1406614. doi: 10.3389/fcvm.2024.1406614 (PMC11176425; doi:10.3389/fcvm.2024.1406614)
Supplement: Supplementary file 2 [file Image2.pdf]

Supplementary Material

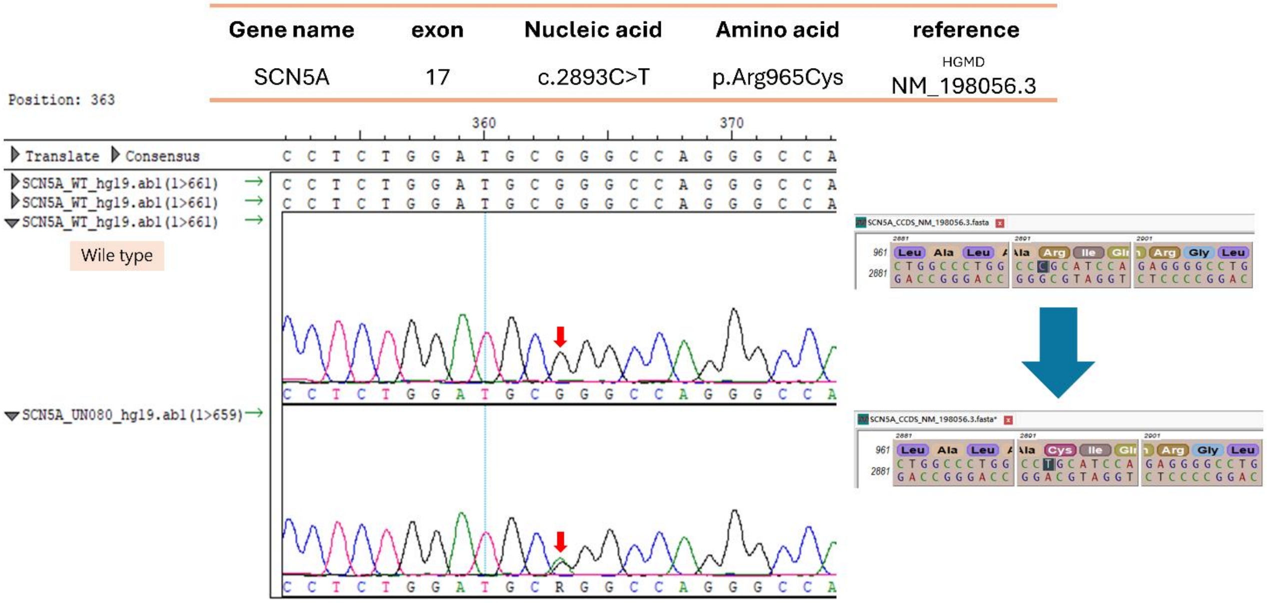

Supplementary Figure 2. Genetic analysis for a case of Brugada syndrome. One heterogeneous missense variant, *SCN5A* c.2893C>T (p.Arg965Cys) was identified (reference transcript: NM_198056.3) in the patient.
